# Genome-wide DNA methylation profiles altered by *Helicobacter pylori* in gastric mucosa and blood leukocyte DNA

**DOI:** 10.18632/oncotarget.9469

**Published:** 2016-05-19

**Authors:** Yang Zhang, Xin-ran Zhang, Jong-lyul Park, Jong-hwan Kim, Lian Zhang, Jun-ling Ma, Wei-dong Liu, Da-jun Deng, Wei-cheng You, Yong-sung Kim, Kai-feng Pan

**Affiliations:** ^1^ Key Laboratory of Carcinogenesis and Translational Research (Ministry of Education/Beijing), Department of Cancer Epidemiology, Peking University Cancer Hospital and Institute, Beijing, China; ^2^ Medical Genomic Research Center, Korea Research Institute of Bioscience and Biotechnology, Daejeon, Korea; ^3^ Department of Medical Oncology, Healthy Bureau of Linqu County, Shandong, China; ^4^ Department of Cancer Etiology, Peking University Cancer Hospital and Institute, Beijing, China

**Keywords:** H. pylori, gastric mucosa, blood leukocyte, methylation array

## Abstract

**Purpose:**

To investigate *Helicobacter pylori (H.pylori)* associated genome-wide aberrant methylation patterns in gastric mucosa and blood leukocyte DNA, a population-based study was conducted in Linqu County.

**Results:**

A total of 3000 and 386 CpGs were differentially methylated after successful *H.pylori* eradication in gastric mucosa and blood leukocyte DNA respectively, and 17 were the same alteration trend in the both tissues. The differentially methylated CpGs were located more frequently in promoters or CpG islands for gastric mucosa and gene body or open sea for blood leukocyte DNA. In eradicated gastric mucosa, the hypermethylated CpGs were enriched across inflammatory pathways, while the hypomethylated CpGs in tube morphogenesis, development and so on. The final validation found lower SPI1, PRIC285 and S1PR4 methylation levels in *H.pylori* positive subjects by case-control comparison, and increased methylation levels in *H.pylori* eradicated gastric mucosa by self-comparison. The Cancer Genome Atlas (TCGA) database analysis suggested that the up-regulation of the three genes by hypomethylation might be associated with gastric carcinogenesis.

**Experimental Design:**

Infinium HumanMethylation 450K BeadChip was used to compare methylation profiles prior to and after eradication treatment. The methylation levels of identified candidate differentially methylated genes before and after *H.pylori* eradication were further validated by two stages (Stage I: self-comparison of 16 subjects before and after anti-*H.pylori* treatment; Stage II: case-control comparison of 25 *H.pylori* positive and 25 negative subjects and self-comparison of 50 anti-*H.pylori* treated subjects).

**Conclusions:**

Novel *H.pylori* associated aberrant methylated genes were identified across the whole genome both in gastric mucosa and blood leukocyte DNA.

## INTRODUCTION

Identified as a class I carcinogen [1], *Helicobacter pylori (H. pylori)* may play important roles in the multi-stage process of gastric carcinogenesis [2]. Our previous studies in a high-risk area of gastric cancer (GC) revealed that *H. pylori* infection increased the risks of GC and precancerous gastric lesions [3, 4], and eradication treatment inhibited or reversed gastric lesion progression [5, 6].

The aberrant epigenetic alterations induced by reactive oxygen species (ROS) and nitric oxide (NO) after *H. pylori* infection in gastric mucosa may serve as one of the main mechanisms in carcinogenesis [7, 8]. For example, hypermethylation of tumor suppressor genes *Cyclin-dependent kinase inhibitor 2A* (*CDKN2A*) [9], *Cadherin 1* [10] and *Runt-related transcription factor 3* (*RUNX3*) [11] was found not only in GC but also in *H. pylori* infected precancerous gastric lesions [12]. Accumulating genome-wide analyses were performed to describe the large-scale characteristics in GC [13], while little is known about the methylation profiles specific for *H. pylori* infection.

Previously, identification of epigenetic biomarkers was mainly focused on tissue samples, however, an increasing number of studies are extracting DNA from non-invasive body fluids such as peripheral blood [14]. Aberrant methylation of blood leukocyte DNA was first reported in lung cancer [15]. Our population-based study also found that the methylation levels of *Insulin-like growth factor 2* (*IGF2*) and *Tumor suppressor candidate 3* (*TUSC3*) in blood leukocytes were significantly increased ahead of clinical diagnosis, which may serve as early biomarkers for GC [16]. Further study showed that the presence of *H. pylori* antibodies was associated with a higher methylation level of *TUSC3* in blood leukocytes, suggesting that the methylation status of peripheral blood may also be affected by *H. pylori* infection [17].

Interestingly, the evidences for origin of blood leukocyte DNA methylation and the relationship with target tissue are still controversial [18]. At present study, we compared the genome-wide methylation profiles prior to and after *H. pylori* eradication in blood leukocytes as well as in gastric mucosa, and further systematically selected and validated novel *H. pylori* associated differentially methylated CpGs/genes in gastric mucosa and/or in blood leukocytes.

## RESULTS

### Comparison of genome-wide methylation profiles between gastric mucosa and blood leukocyte DNA

**Figure 1:**
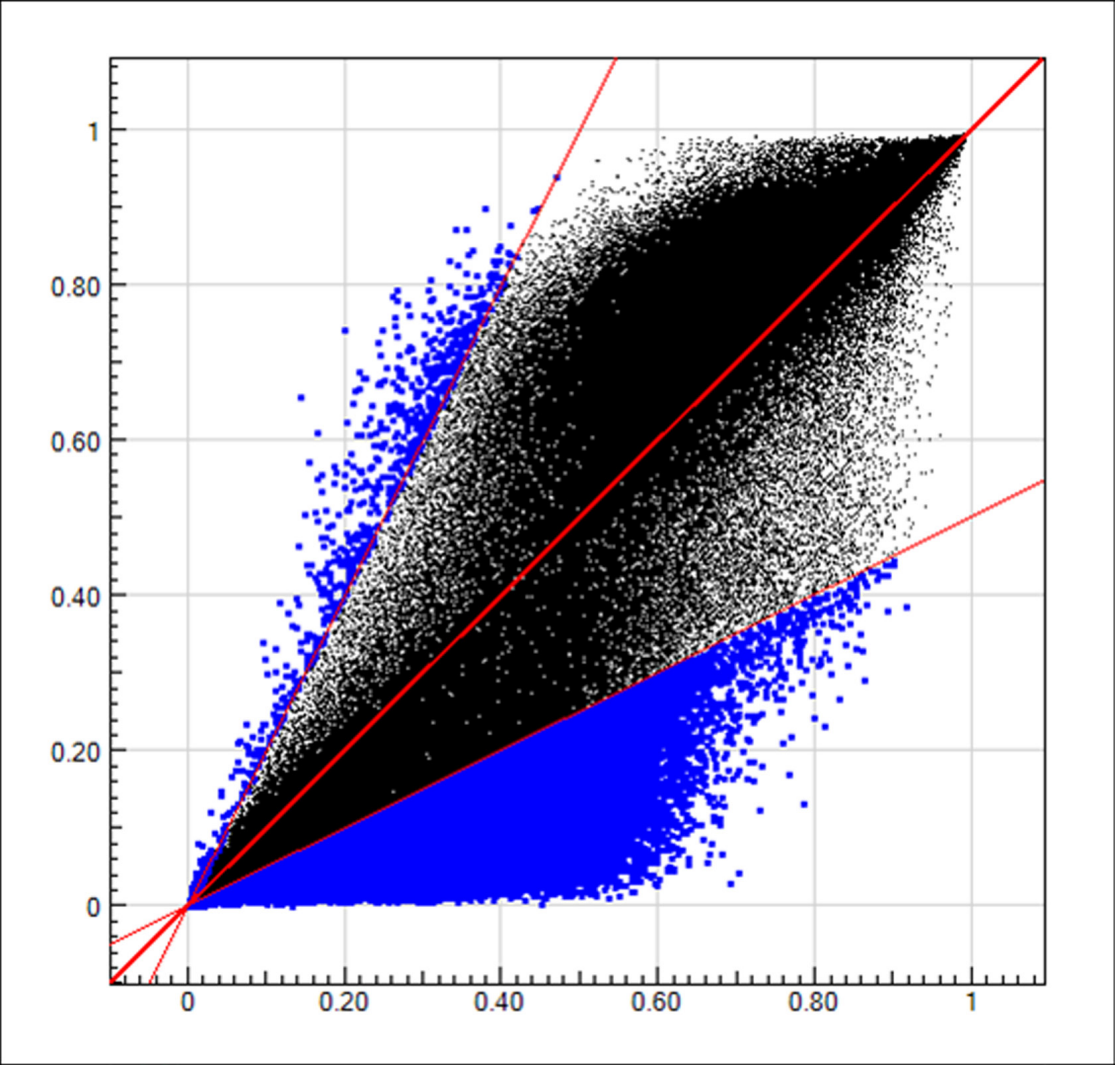
The methylation levels in gastric mucosa and blood leukocyte DNA The blue dots in upper left region represent the CpGs with (methylation level in gastric mucosa)/(methylation level in blood leukocytes) ≤ 0.5. The blue dots in lower right region represent the CpGs with (methylation level in gastric mucosa)/(methylation level in blood leukocytes) ≥ 2. The black dots in the middle region represent the CpGs with (methylation level in gastric mucosa)/(methylation level in blood leukocytes) > 0.5 and < 2.

General characteristics of the 8 subjects for methylation array detection were described in Supplementary Table S1. For correlation analysis between methylation levels in gastric mucosa and blood leukocytes, the baseline *H. pylori* positive samples of the 6 successfully and 2 unsuccessfully eradicated subjects were combined and calculated the average methylation levels (β values). The methylation levels of more than 480,000 CpGs in the Infinium methylation array were highly correlated between gastric mucosa and blood leukocyte DNA with the *R*^2^ as 0.89 (Figure 1).

### Selection of differentially methylated CpGs before and after *H. pylori* eradication

**Figure 2:**
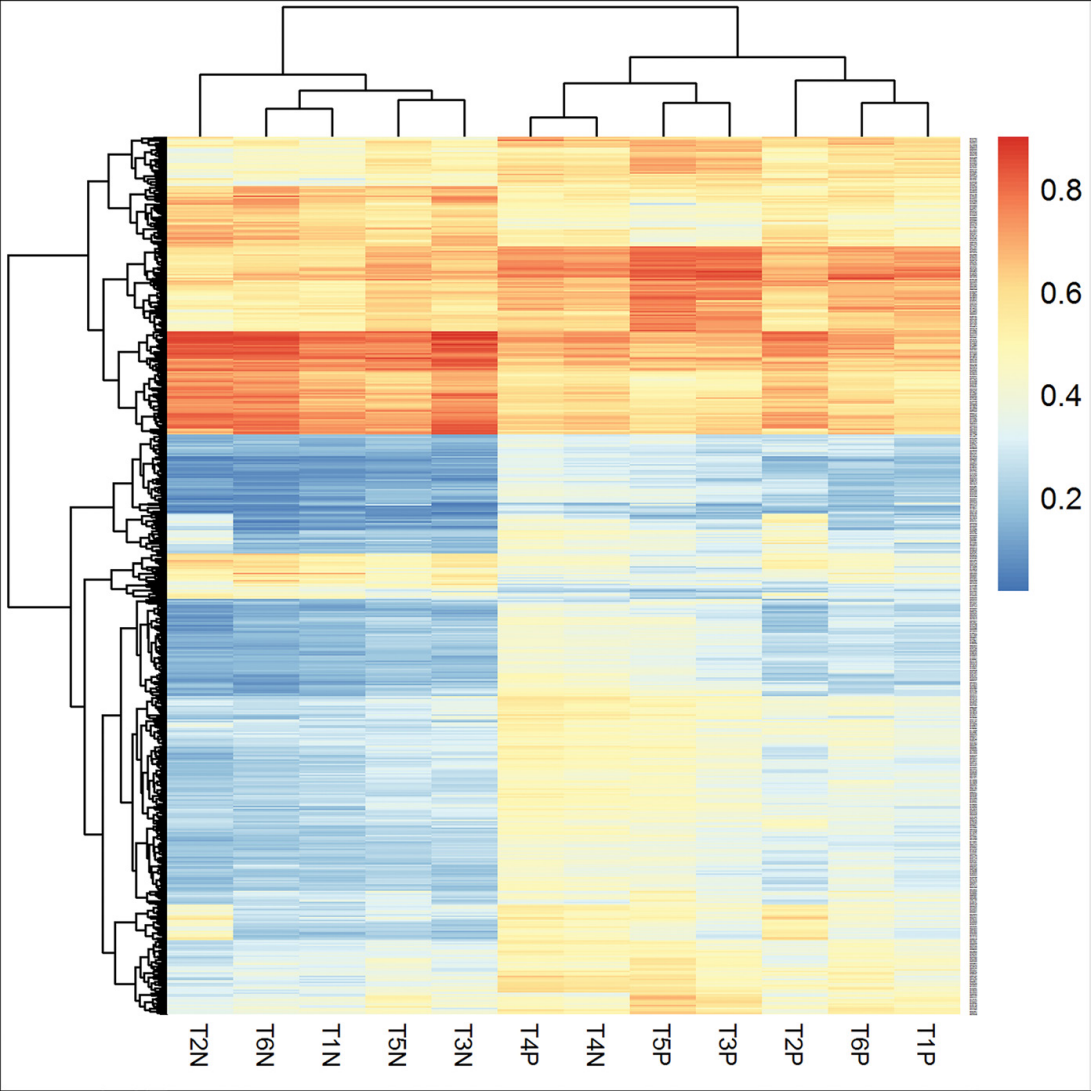
Hierarchical cluster analysis of the differentially methylated CpGs in gastric mucosa before and after *H. pylori* successful eradication “T*P” represents *H. pylori* positive gastric mucosa before eradication and “T*N” represents *H. pylori* negative gastric mucosa after successful eradication. The methylation levels of the significant CpGs were distinguished between *H. pylori* positive gastric mucosa prior to treatment and negative samples after eradication. Methylation level is shown as a continuous variable (0 to 1) from a blue to red color.

To identify differentially methylated CpGs in gastric mucosa, we selected 3026 significant CpGs (|Δβ| ≥ 10%, *P* < 0.05) prior to and after successful *H. pylori* eradication. Among them, 26 were excluded for overlapping with the significant CpGs in 2 unsuccessfully eradicated subjects. In the 3000 selected *H. pylori* associated differentially methylated CpGs (covering 1699 genes), 1570 (52.3%) were hypomethylated (Δβ ≥ 10%) and 1430 (47.7%) were hypermethylated (Δβ ≤ −10%) after eradication. Figure 2 showed a hierarchical cluster analysis of the 568 top significant CpGs (*P* < 0.01) in 6 pairs of gastric mucosa samples prior to and after successful *H. pylori* eradication. The methylation levels of these significant CpGs were distinguished between *H. pylori* positive gastric mucosa prior to treatment and negative samples after eradication.

Using the same strategy, only 2 significant CpGs with |Δβ| ≥ 10% (*P* < 0.05) were found in blood leukocyte DNA. To select more potential differentially methylated CpGs in blood leukocytes, we used a smaller cut-off |Δβ| as 5% and found a total of 386 differentially methylated CpGs (covering 282 genes) in blood leukocytes (|Δβ| ≥ 5%, *P* < 0.05), with 71 (18.4%) hypomethylated and 315 (81.6%) hypermethylated after *H. pylori* eradication. Furthermore, to select more potential common candidate significant CpGs overlapped between gastric mucosa and blood leukocytes, the smaller cut-off |Δβ| of 5% in blood leukocytes was used. A total of 33459 (from gastric mucosa) and 386 (from blood leukocytes) significant CpGs (|Δβ| ≥ 5%, *P* < 0.05) were compared, and 17 CpGs (involving 11 genes) were overlapped with the same trends of methylation changes after eradication between gastric mucosa and blood leukocyte DNA (Table 1).

**Table 1:** Differentially methylated CpGs overlapped between blood leukocytes and gastric mucosa

ID	Average Δβ in blood leukocyte	Average Δβ in gastric mucosa	Chr.	CpG site	Gene name	Location with gene	Location with CGI
cg12643083	−7.38%	−9.24%	1	159743172	FCGR2A	Body	
cg22813165	−6.69%	−5.46%	19	40794451	HAUS5	TSS1500	N_Shore
cg10180440	−6.58%	−5.49%	10	102315476			S_Shelf
cg23772352	−6.15%	−6.65%	12	124909736			N_Shelf
cg05018460	−6.14%	−5.93%	15	78475133			
cg05025071	−5.83%	−6.71%	19	6838530	EMR1	TSS200	
cg13686044	−5.63%	−6.25%	17	14677092			
cg25025181	−5.58%	−10.32%	1	244445133	SMYD3	Body	
cg22534374	−5.45%	−5.54%	1	199778233			S_Shelf
cg08904369	−5.39%	−5.94%	6	167624178	UNC93A	TSS1500	
cg06518781	−5.25%	−5.55%	19	22495123			S_Shelf
cg14183176	−5.13%	−7.34%	3	123982893	HSPBAP1	Body	
cg24202468	−5.12%	−7.61%	13	97993340	STK24	Body	
cg01711344	−5.10%	−11.86%	11	67526980	UNC93B1	Body	N_Shore
cg05590451	5.57%	8.15%	16	20798035	DCUN1D3	5′UTR	
cg01785046	6.43%	5.16%	7	91348383	MTERF	TSS1500	S_Shore
cg06522206	6.73%	7.10%	12	116135682	NOS1	3′UTR	

**Figure 3:**
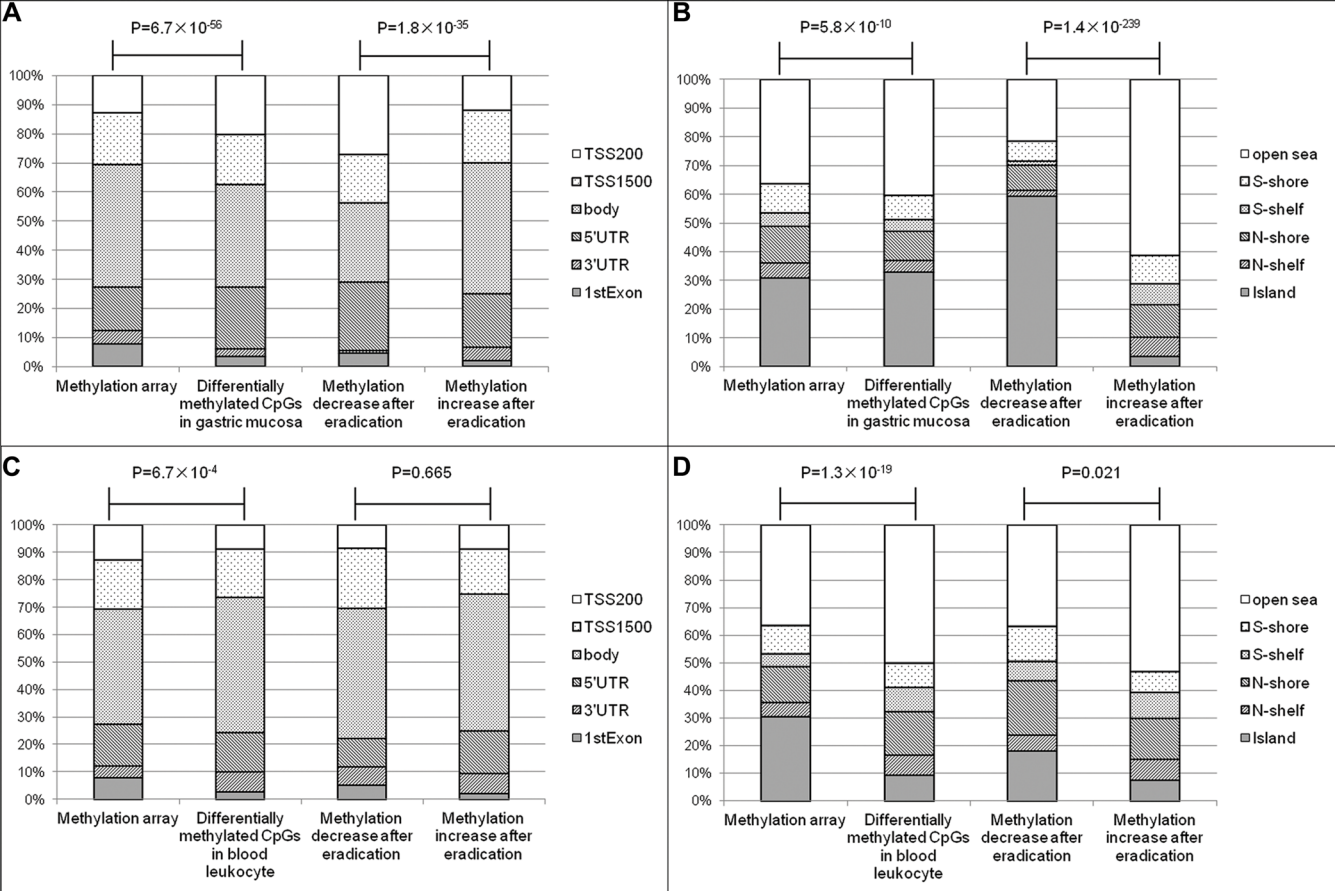
The relationships between differentially methylated CpGs and genes or CGIs (**A**) The relationships between differentially methylated CpGs of gastric mucosa and genes. (**B**) The relationships between differentially methylated CpGs of gastric mucosa and CGIs. (**C**) The relationships between differentially methylated CpGs of blood leukocytes and genes. (**D**) The relationships between differentially methylated CpGs of blood leukocytes and CGIs.

According to the manufacturer information, the CpGs in Infinium methylation array were located 7.8% in the 1^st^ exon, 15.0% in 5′ un-translated regions (UTR), 12.9% in 200 bp upstream of transcription start site (TSS200), 17.9% in TSS1500, 4.4% in 3′UTR and 42.0% in gene body, respectively. Comparing to the array distribution, the significant CpGs in gastric mucosa were found more frequently in 5′UTR (21.2%) and TSS200 (20.4%), and less frequently in gene body (35.2%), *P* = 6.7 × 10^−56^. Furthermore, the hypomethylated CpGs were found more frequently in 5′UTR (23.6% *vs.* 18.2%) and TSS200 (27.2% *vs.* 12.0%) than hypermethylated ones, *P* = 1.8 × 10^−35^ (Figure 3A). In contrast with the tendency in gastric mucosa, the significant CpGs in blood leukocytes were located less frequently in 1^st^ exon (2.8%) and TSS200 (8.8%), and more frequently in gene body (49.5%), *P* = 6.7 × 10^−4^. In addition, there was no statistical difference between the distributions of hypo- and hypermethylated CpGs after eradication in blood leukocyte DNA, *P* = 0.665 (Figure 3C).

Because aberrant methylation has been frequently found in CpG islands (CGIs), shores (regions up to 2 kb from the islands) and shelves (2–4 kb from the islands), we further investigated the relationship between the differentially methylated CpGs and CGIs by comparing with the distribution of methylation array [30.9% in CGI, 5.1% in N (north)-shelf, 4.6% in S (south)-shelf, 13.0% in N-shore, 10.1% in S-shore and 36.3% in open sea]. For gastric mucosa, the significant CpGs were located more frequently in CGIs (32.8%), *P* = 5.8 × 10^−10^, and hypomethylated CpGs were found more frequently in CGIs (59.4%) than hypermethylated CpGs (3.6%), *P* = 1.4 × 10^−239^ (Figure 3B). For the significant CpGs in blood leukocytes, only 9.6% were found in CGIs, which was much less than the composition of methylation array, *P* = 1.3 × 10^−19^. The hypomethylated CpGs were located more frequently in CGI (18.3%) than hypermethylated CpGs (7.6%) after eradication, *P* = 0.021 (Figure 3D).

### Gene ontology and pathway categories of differentially methylated CpGs after *H. pylori* eradication

For Gene ontology (GO) and Kyoto Encyclopedia of Genes and Genomes (KEGG) pathway analyses, we selected the differentially methylated CpGs located from TSS to TSS1500 and in CGIs. The correlation between methylation levels at the selected CpGs and their target gene expressions was analyzed in 230 GC tissues collected from The Cancer Genome Atlas (TCGA) dataset (http://cancergenome.nih.gov/) by Illumina HumanMethylation 450 K BeadChip array and RNA-sequencing platform. A total of 294 hypo- and 51 hypermethylated CpGs after successful eradication were negatively associated with their target gene expressions (*R* ≤ −0.2, *P* < 0.05), which were enrolled in the GO and KEGG pathway analyses.

After successful eradication in gastric mucosa, the hypermethylated CpGs were associated with the inflammatory process and pathways, such as positive regulation of immune system process (Supplementary Table S2), B and T cell receptor signaling pathway and leukocyte transendothelial migration (Supplementary Table S3). While, the hypomethylated CpGs were enriched in tube morphogenesis and development, embryonic morphogenesis and development, homophilic cell adhesion, response to hormone stimulus, epithelium development and so on (Supplementary Table S2). For the differentially methylated CpGs in blood leukocyte DNA, GO and KEGG pathway analyses showed the *P* values to be more than 0.05 and not significantly enriched.

### Stage I validation of candidate differentially methylated genes

From the list of top differentially methylated CpGs located in 5′UTR, TSS200 or 1^st^ exon (Supplementary Table S4), 15 and 3 CpGs (covering 12 and 3 genes) were selected from gastric mucosa and blood leukocyte DNA for Stage I validation. From the 11 candidate overlapped significant genes between gastric mucosa and blood leukocytes, only *MTERF* and *HAUS5* genes were detected in Stage I, because the involving significant CpGs were near to the promoter (TSS1500) and CGIs (shore). The CpGs in the promoters of the candidate genes were amplified using CpG-free primer sets, which were analyzed quantitatively by denaturing high performance liquid chromatography (DHPLC) for methylation levels in the two-stage validation.

A total of 16 subjects were selected for Stage I validation, including 8 successfully and 8 unsuccessfully *H. pylori* eradicated cases. The baseline gastric lesions included 7 superficial gastritis, 4 chronic atrophic gastritis, 3 intestinal metaplasia and 2 mild dysplasia. Among 12 candidate genes from gastric mucosa, the methylation levels of *SPI1* and *PRIC285* were increased from 34.9% to 53.8% (*P* = 0.022) and 35.5% to 50.1% (*P* = 0.024) after successful eradication, respectively (Table 2). While in unsuccessful eradication group, no statistical methylation change was found for these genes. In addition, although the *P* values showed no statistical differences (0.132 and 0.069), *S1PR4* and *CELSR3* were also selected for Stage II validation because of the methylation increasing of 15.5% and decreasing of 9.6% after eradication, respectively.

**Table 2:** Stage I self-comparison validation for candidate differentially methylated genes

Gene name	Average methylation before successful eradication	Average methylation after successful eradication	Methylation difference after successful eradication	*P*^1^ value	Average methylation before unsuccessful eradication	Average methylation after unsuccessful eradication	Methylation difference after unsuccessful eradication	*P*^1^ value	*P*^2^ value
Gastric mucosa (M)									
NEU1	8.1%	8.2%	−0.1%	0.974	6.1%	5.5%	0.6%	0.700	0.877
PLEKHG6	35.5%	26.5%	9.0%	0.632	51.2%	34.6%	16.6%	0.395	0.769
PRIC285	35.5%	50.1%	−14.6%	0.024	27.2%	32.8%	−5.6%	0.062	0.137
S1PR4	7.3%	22.8%	−15.5%	0.132	7.1%	7.4%	−0.3%	0.963	0.175
SPI1	34.9%	53.8%	−18.9%	0.022	23.2%	22.0%	1.2%	0.726	0.015
SYNM	1.8%	1.7%	0.1%	0.982	2.4%	4.5%	−2.1%	0.566	0.587
ARMC4	4.0%	0.9%	3.1%	0.086	2.9%	4.9%	−2.0%	0.531	0.163
GP1BB	1.6%	2.2%	−0.6%	0.316	2.9%	2.1%	0.8%	0.574	0.349
KANK3	3.4%	10.0%	−6.6%	0.110	5.7%	5.1%	0.6%	0.768	0.100
KCNQ3	8.4%	6.5%	1.9%	0.568	3.0%	2.0%	1.0%	0.744	0.823
CELSR3	9.8%	0.2%	9.6%	0.069	3.8%	2.3%	1.5%	0.665	0.194
FOXQ1	47.5%	49.4%	−1.9%	0.899	46.8%	43.2%	3.6%	0.710	0.755
Blood leukocyte (B)									
GNAS	40.5%	47.6%	−7.1%	0.549	49.1%	51.9%	−2.8%	0.842	0.817
BCOR	64.8%	66.0%	−1.2%	0.795	93.8%	94.4%	−0.6%	0.507	0.896
LTBR	0.3%	1.7%	−1.4%	0.375	1.5%	1.7%	−0.2%	0.862	0.468
Overlapped between gastric mucosa (M) and blood leukocyte(B)									
MTERF(M)	5.9%	2.3%	3.6%	0.057	3.9%	5.0%	−1.1%	0.284	0.023
MTERF(B)	2.3%	0.8%	1.5%	0.107	2.8%	1.2%	1.6%	0.043	0.933

1 Paired *t* test, comparing the methylation level before and after *H. pylori* eradication in successful and unsuccessful groups.

2 *t* test, comparing the average methylation differences between successful and unsuccessful eradication groups.

For the 3 candidate genes from blood leukocytes, no significant methylation change was found either in successful or unsuccessful eradication group (Table 2). As a candidate gene overlapped between gastric mucosa and blood leukocytes, *MTERF* was hypomethylated both in gastric mucosa (5.9% to 2.3%) and in blood leukocytes (2.3% to 0.8%) after eradication, although the *P* values (0.057 and 0.107) showed no statistical significance (Table 2). While for *HAUS5*, no positive result was found in gastric mucosa and blood leukocyte DNA.

### Stage II validation of candidate differentially methylated genes

In Stage II validation, 25 *H. pylori* positive and 25 negative subjects were selected for case-control comparison and 50 pairs of gastric mucosa samples before and after eradication treatment were enrolled for self-comparison, respectively. For the case-control comparison, 4 candidate genes (*SPI1, PRIC285, S1PR4* and *CELSR3*) were selected from Stage I validation. As shown in Supplementary Table S5, the frequency of chronic atrophic gastritis was higher in *H. pylori* positive (52.0%) than in negative groups (16.0%, *P* = 0.021). No significant difference in age, sex, cigarette smoking, and alcohol consumption was found between two groups.

The univariate analysis showed significant lower methylation levels of *SPI1, PRIC285* and *S1PR4* in *H. pylori* positive than in negative groups (*SPI1*: 39.7% *vs.* 81.1%, *P* < 0.001; *PRIC285*: 44.5% *vs.* 74.6%, *P* = 0.002; *S1PR4*: 34.8% *vs.* 66.7%, *P* = 0.002). Using the methylation medians in negative group (*SPI1*: 89.9%, *PRIC285*: 79.5%, *S1PR4*: 80.3%) as cut-off values, we divided the methylation status into hyper- and hypo-methylation groups and found that *SPI1* and *S1PR4* methylation were decreased significantly in *H. pylori* positive subjects [*SPI1*: Odd ratio (OR), 0.12; 95% confidence interval (CI), 0.02–0.70, *S1PR4*: OR, 0.15; 95%CI, 0.03–0.75] (Table 3).

**Table 3:** Associations between methylation status and *H. pylor**i*** infection in gastric mucosa in Stage II case-control validation

	*H. pylori* negative *n* (%)	*H. pylori* positive *n* (%)	*P* value	OR^a^	95% CI^a^
SPI1 (cut-off value 89.9%)					
Hypermethylated	12 (48.0)	2 (8.0)	0.018	0.12	0.02–0.70
Hypomethylated	13 (52.0)	23 (92.0)			
PRIC285 (cut-off value 79.5%)					
Hypermethylated	12 (48.0)	5 (20.0)	0.238	0.41	0.09–1.81
Hypomethylated	13 (52.0)	17 (68.0)			
Missing		3 (12.0)			
S1PR4 (cut-off value 80.3%)					
Hypermethylated	13 (52.0)	3 (12.0)	0.021	0.15	0.03–0.75
Hypomethylated	10 (40.0)	20 (80.0)			
Missing	2 (8.0)	2 (8.0)			
CELSR3 (cut-off value 0%)					
Methylated	8 (32.0)	9 (36.0)	0.969	0.97	0.24–3.97
Unmethylated	15 (60.0)	14 (56.0)			
Missing	2 (8.0)	2 (8.0)			

a Unconditional logistic regression analysis, adjusted for age, sex, smoking, drinking and pathologic diagnosis.

Among 50 self-comparison subjects, 37 were successfully *H. pylori* eradicated. The distribution of age, gender, smoking, drinking and baseline pathology showed no significant difference between the two groups (Supplementary Table S6). After successful eradication, the methylation levels of *SPI1*, *PRIC285* and *S1PR4* were increased significantly (*SPI1*: 34.6% to 75.2%, *P* < 0.001; *PRIC285*: 46.8% to 70.4%, *P* < 0.001; *S1PR4*: 12.9% to 26.7%, *P* = 0.003). In unsuccessful group, *S1PR4* and *PRIC285* genes showed no remarkable change (17.0% to 14.0%, *P* = 0.664; 44.8% to 45.0%, *P* = 0.979, respectively), while *SPI* methylation level decreased after treatment (59.0% to 33.1%, *P* = 0.027). The methylation level changes of the three genes showed significant differences between successful and unsuccessful eradication groups (all *P* < 0.05).

### The clinical relevance of *SPI1*, *PRIC285*, *S1PR4* methylation and correlation with expression in GC by TCGA database

**Figure 4:**
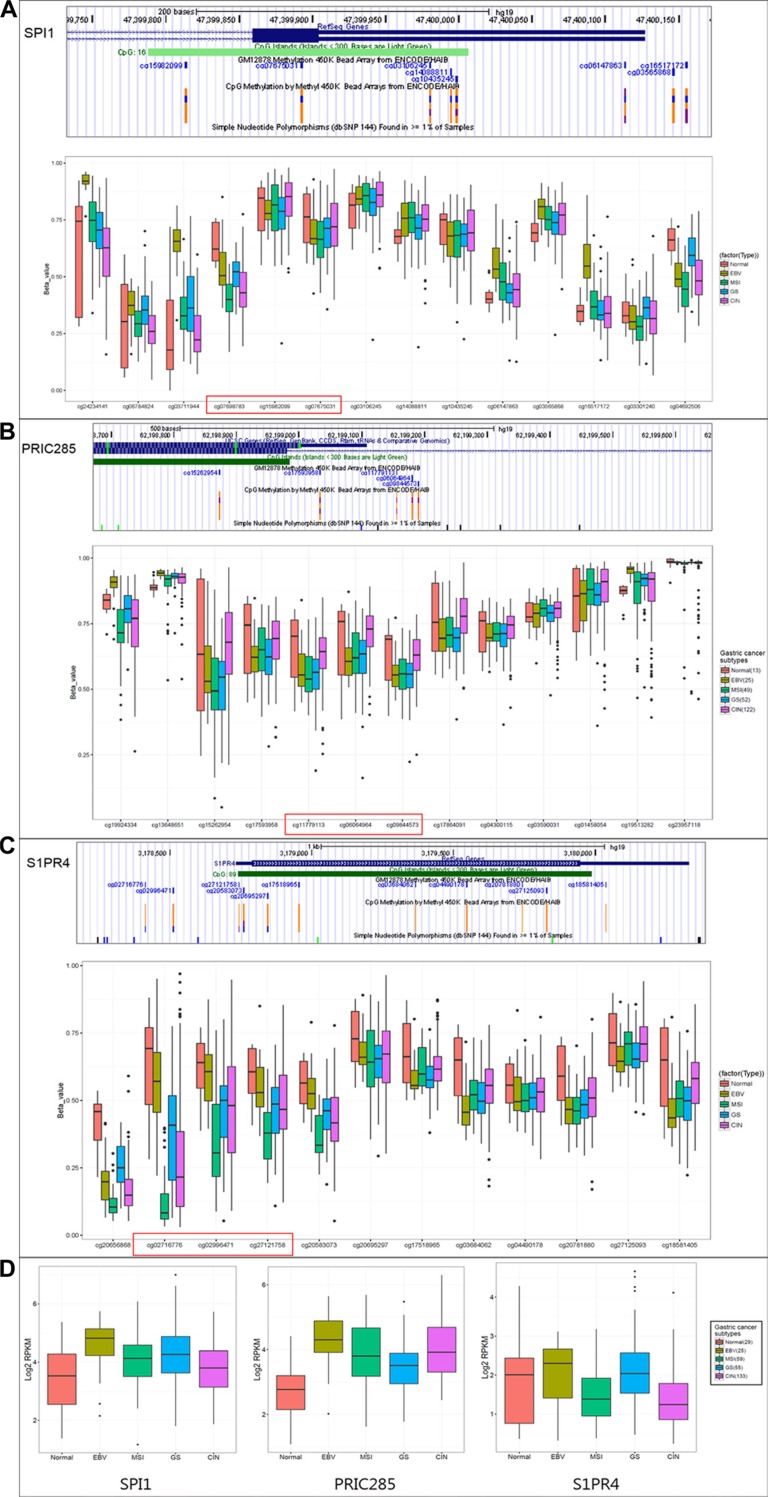
The clinical relevance of *SPI1, PRIC285, S1PR4* methylation and correlation with expression in GC by TCGA database The promoter methylation status (**A**, **B**, **C**) and RNA expression levels (**D**) of *SPI1*, *PRIC285* and *S1PR4* genes in TCGA public genome database of 29 normal and 230 GC subjects, which were divided into four subtypes (EBV: Epstein–Barr virus-positive; MSI: microsatellite instability high status; GS: genomically stable; CIN: chromosomal instability).

To describe a clinical relevance of *SPI1*, *PRIC285* and *S1PR4* genes, we investigated their promoter methylation status and expression levels in TCGA public genome database (http://cancergenome.nih.gov/) of 29 normal and 230 gastric tumors, which were divided into four subtypes by molecular classification [19]. The methylation levels of CpGs on promoters (cg07698783, cg15982099, cg07675031 on *SPI1*; cg11779113, cg06064964, cg09844573 on *PRIC285* and cg02716776, cg02996471, cg27121758 on *S1PR4*) were dramatically lower in the majority of GCs than in normal tissues (Figure 4A–4C).

From RNA-sequencing data of TCGA, we also found that the expressions of *SPI1* or *PRIC285* were increased in all types of GC compared to normal tissues and *S1PR4* was increased in Epstein-Barr virus-positive and genomically stable subtypes (Figure 4D), suggesting that up-regulation of these genes in GCs might be associated with the decrease of promoter CpGs methylation.

## DISCUSSION

Using Infinium HumanMethylation 450 K BeadChip, a comprehensive assessment of genome-wide epigenetic alterations was conducted by comparing blood leukocyte DNA as well as gastric mucosa DNA prior to and after anti-*H. pylori* treatment, respectively. Correlations were evaluated firstly between the methylation levels of gastric mucosa and blood leukocyte DNA, and novel *H. pylori* associated differentially methylated CpGs/genes were selected and validated.

Recently, some large-scale comparisons of DNA methylation profiles between blood leukocytes and matched target tissues (such as adipose and brain) suggested that the concordant genes might be tissue specific [20–22]. The present comprehensive array analysis enabled us to compare the methylation levels between blood leukocytes and gastric mucosa systematically for the first time. We found the methylation levels were highly correlated between the two kinds of tissues at baseline (*R*^2^ = 0.89), which implied that blood leukocyte DNA methylation might serve as a potential surrogate biomarker for the methylation status of gastric mucosa.

Recent high-throughput technologies enabling global DNA methylome analysis have been used in various cancers to elucidate epigenetic mechanisms [23, 24]. In 2012, a study found that 11,740 CpGs were differentially methylated in GC tissues with 83% hypermethylated using Infinium 27 K methylation array [13], which covered a total of 27,578 CpGs and 14,495 gene promoters in the human genome. In our present study, the CpGs were not limited to the promoter and CGIs and also involved in intragenic regions and gene deserts by Infinium 450 K methylation array. The comparison results found 3000 differentially methylated CpGs with 52.3% hypomethylated and 47.7% hypermethylated after eradication in gastric mucosa, suggesting that *H. pylori* infection may be associated with hypermethylation as well as hypomethylation in the early stage of gastric lesion evolution.

The distributions of differentially methylated CpGs may provide important information about epigenetic mechanism in human carcinogenesis. For example, “tandem control” mechanism was suggested for epigenetic regulation, in which promoter hypermethylation and gene body hypomethylation may lead to repression, or promoter hypomethylation and gene body hypermethylation may lead to activation [13]. In our study, *H. pylori* infection might more likely induce genes repression by hypermethylation of promoter and hypomethylation of gene body in gastric mucosa. In addition, several tumor studies showed that significantly hypermethylated CpGs were found more frequently at CGIs, while most hypomethylated CpGs were located at open sea regions [25–27]. Our current findings for *H. pylori* associated differentially methylated CpGs were consistent with the above results, which suggested the epigenetic alterations induced by *H. pylori* might be related to gastric carcinogenesis.

To date, few studies have examined the association between methylation levels in blood leukocyte DNA and risk of GC [17, 28]. We found 386 differentially methylated CpGs (|Δβ| ≥ 5%) that may be associated with *H. pylori* infection in blood leukocyte DNA. Compared to those in gastric mucosa, the number of the differentially methylated CpGs in blood leukocytes was much less, with more hypermethylation (81.6%) and less hypomethylation (18.4%). On the other hand, the significant CpGs from blood leukocyte DNA were located more frequently at gene body and open sea region, which was different with those from gastric mucosa. In addition, the 17 overlapped significant CpGs between gastric mucosa and blood leukocyte DNA were also found outside the promoter and CGIs regions. These distribution discrepancies and higher frequency of hypermethylation in blood leukocyte DNA after eradication were consistent with the previous findings of genomic hypomethylation in *H. pylori* positive subjects [29, 30], suggesting that *H. pylori* associated epigenetic alterations in blood leukocyte DNA might be more related with global hypomethylation rather than the aberrant methylation of specific promoter CGI loci.

GO and KEGG pathway analyses of the differentially methylated CpGs in gastric mucosa showed that hypermethylated CpGs in promoter occurred at immune response process and pathways, consisting with the active process of the inflammation after *H. pylori* infection. Our study also showed that hypomethylated CpGs in promoter were enriched in various biological processes and molecular functions. Among them, cell-cell adhesion was noticed for the vital roles to inhibit individual epithelial cell motility and to provide homeostatic gastric tissue architecture. Being a crucial factor for cell-cell adhesion function, E-cadherin (encoded by *CDH1* gene) was found to be silenced by *H. pylori* infection and the average methylation level was decreased 10.1% after eradication in our present study.

The current array-based methylation profiling identified a large number of *H. pylori* associated differentially methylated CpGs in gastric mucosa and blood leukocyte DNA. In the final larger sample size validation, *SPI1, PRIC285* and *S1PR4* methylation levels in gastric mucosa were decreased when *H. pylori* positive subjects were compared with negative cases, and increased after successful eradication by self-comparison. The consistent results from the case-control and self-comparison studies suggested that methylation alterations of *SPI1, PRIC285* and *S1PR4* in gastric mucosa might be associated with *H. pylori* infection. We also identified several differentially methylated CpGs overlapped between gastric mucosa and blood leukocyte DNA, although a negative validation result was found after the detection of overall methylation status in promoter region by DHPLC rather than the methylation levels of specific CpGs by probes in methylation array.

According to the reports, SPI1 is important for the transcriptional regulation of interleukin-1β, involving in inflammatory and immunoregulatory process [31]. PRIC285 acts as a nuclear receptor coactivator by stimulating peroxisome proliferators-activated receptor α (PPARα)-mediated transcription [32], and was down-regulated in the leukocyte fraction of epithelial ovarian cancer subjects [33]. S1PR4 is one of the five G protein-coupled receptors of sphingosine-1-phosphate (S1P) [34], which has received much attention for its potential roles in tumorigenesis and therapies [35, 36]. No epigenetic regulation mechanism was reported for *SPI1*, *PRIC285* or *S1PR4* genes in gastric lesions or GC, until our methylation array screening and validations, together with TCGA database analysis, suggested that the up-regulation of the three genes by the decrease of promoter methylation might be associated with *H. pylori* infection and gastric carcinogenesis. However, further functional studies are still needed to verify the relationships and mechanisms between the aberrant methylation and expression in gastric mucosa or *H. pylori* infection.

The major strengths of our study lie in a clinical trial design by comparing the methylation levels prior to and after *H. pylori* eradication, which can control other confounders, such as age, gender, environmental and exposure factors. To exclude the effects on methylation alterations by intervention drugs, we also compared the methylation changes between successful and unsuccessful eradication groups. The correlations between the methylation levels in gastric mucosa and blood leukocyte DNA were evaluated in large-scale for the first time, and provided important clues for surrogate biomarker identification in blood leukocyte DNA. A limitation in our study may be the fact that the case number for validation study was small. For the subsequent validation of aberrant methylation genes in blood leukocyte DNA, a new candidate gene selection strategy will be needed because of the different methylation alteration trend and different distribution of significant CpGs between gastric mucosa and blood leukocyte DNA.

In summary, we have comprehensively characterized genome-wide DNA methylation patterns in gastric mucosa and blood leukocyte DNA and identified a large subset of candidate CpGs/genes associated with *H. pylori* infection. Several differentially methylated CpGs were overlapped between gastric mucosa and blood leukocyte DNA, although a negative validation result was currently shown. The validations in gastric mucosa identified three novel potential aberrant methylation genes (*SPI1, PRIC285* and *S1PR4*) associated with *H. pylori*, which still need to confirm in a larger sample size investigation and further functional study.

## MATERIALS AND METHODS

### Study population

A large intervention trial to prevent GC by *H. pylori* eradication was launched in March 2011 and completed in September 2013 in Linqu County [37]. During this period, an endoscopic screening project was conducted in Linqu, in which 33 and 108 *H. pylori* positive subjects participated in the both projects in 2012 and 2013, respectively. For our current study, blood and fresh gastric tissue samples were collected in the endoscopic screening project prior to anti-*H. pylor*i treatment at baseline and a repeat endoscopy examination at six months after the treatment. For genome-wide methylation profiling and Stage I validation, we totally selected 16 subjects (8 successfully and 8 unsuccessfully eradicated) from 33 participants in 2012. Among them, 6 successfully and 2 unsuccessfully eradicated subjects with mild gastric lesions were used for methylation profiling, and all of the 16 subjects were enrolled in Stage I validation. For Stage II validation, 25 *H. pylori* positive and 25 negative subjects were selected randomly from the baseline endoscopic screening in 2013 for case-control design. Furthermore, 50 subjects with blood and fresh gastric tissue samples prior to and after treatment were selected at random from 108 participants in 2013 for self-comparison design.

The detailed procedure of endoscopy has been described elsewhere [6]. Briefly, upper endoscopy examinations were conducted by four experienced gastroenterologists. The gastric mucosa was examined and five biopsies were obtained from standard sites of the stomach according to the Updated Sydney System [38]. For the present study, one or two additional biopsies were obtained from the lesser curvature of antrum or angulus adjacent and frozen in liquid nitrogen immediately. All specimens were reviewed by a panel of three pathologists according to the Updated Sydney System [38] and Padova International Classification [39]. The detailed information of *H. pylori* screening and treatment has been reported on our baseline results of large intervention trial [37].

A written informed consent was obtained from each subject and the study was approved by the Institutional Review Board of Peking University Cancer Hospital.

### Genome-wide methylation profiling in gastric mucosa and blood leukocyte

The DNA from gastric mucosa and blood leukocytes was extracted using Dneasy Tissue and Blood kits (Qiagen) and bisulfite-converted using EZ DNA methylation kit (Zymo Research) according to the manufacturer's protocol.

Genome-wide methylation profiling was performed using Infinium HumanMethylation450 BeadChip array (Illumina, San Diego, CA, USA). After whole-genome amplification with 200ng of input bisulfite-converted DNA, the product was fragmented, purified and applied to the BeadChips using Illumina-supplied reagents and conditions. After extension, the array was stained fluorescently, and scanned with an iSan System (Illumina). The data were analyzed by GenomeStudio Methylation Module Software (Illumina). A CpG site was considered to be informative if the sum of the signals for methylated and unmethylated sequence at the CpG site was significantly higher (detection *P* value < 0.05) than signals of the negative control probes on the same array. For each CpG site, the β value reflects the methylation level, which was computed by β = (max (M, 0))/(|U| + |M|+100). A β value of 0–1.0 indicates the percent methylation from 0% to 100%, respectively.

### Identification of differentially methylated CpGs and genes

To select candidate differentially methylated CpGs before and after *H. pylori* eradication, the methylation levels of 484,543 and 484,435 target CpGs in methylation array with detection *P* values ≤ 0.05 were analyzed in 6 successfully eradicated subjects. When the methylation differences before and after eradication were ≥ 10% in gastric mucosa (≥ 5% in blood leukocyte) with *P* values less than 0.05, the CpGs were selected as *H. pylori* associated differentially methylated ones by excluding those overlapped by unsuccessful eradication group. To identify candidate *H. pylori* associated differentially methylated genes for further validation, the genes with top differentially methylated CpGs in 5′UTR, TSS200, exon-1 regions and CGIs were focused on due to their well-known correlations with gene transcription regulation.

### Hot-start PCR and DHPLC analysis

CpG-free universal primer sets and bisulfite-modified DNA were used to amplify the selected candidate genes. The PCR reaction mixture (20 μL) included 20 ng DNA template, 0.5 mmol/L dNTP, 0.5 μmol/L of each primer, and 1.0 U of HotStart Taq DNA polymerase (Qiagen GmbH). The PCR products were then analyzed quantitatively by DHPLC. Hypermethylated and hypomethylated PCR products were separated using a DNASep analytical column (Transgenomic) at the corresponding partial denaturing temperature. The primer sequence and partial denaturing temperature were listed in the Supplementary Table S7. M. SssI-methylated genomic DNA, obtained from blood samples, was used as a positive control. A sample containing a methylated PCR product peak was defined as methylation-positive. The peak areas corresponding to the methylated and unmethylated PCR products were used to calculate the percentage of methylated copies (proportion of hypermethylated copies = methylated peak area/total peak area) for each candidate gene (Supplementary Figure S1A). The methylation of the CpGs was further confirmed using traditional bisulfite sequencing. The bisulfite sequencing results were consistent with the DHPLC analysis (Supplementary Figure S1B).

### Statistical analysis

The student paired *t* test was used to identify the differentially methylated CpGs and genes before and after *H. pylori* eradication by methylation array or DHPLC detections. The Mann-Whitney *U* test was used to compare the methylation level differences between successful and unsuccessful eradication groups. In Stage II case-control validation, *χ*^2^ test was used to analyze the differences in age (separated into two groups according to median age), sex, smoking, drinking and baseline pathological diagnosis between *H. pylori* positive and negative groups. The differences of methylation levels between *H. pylori* positive and negative groups were calculated by Mann-Whitney *U* test. The frequency of hyper- or hypo-methylated candidate genes (according to the median methylation levels in negative group) between *H. pylori* positive and negative groups was compared by unconditional logistic regression, adjusting age, sex, smoking, drinking and baseline pathological diagnosis. All statistical analyses were carried out using Statistical Product and Service Solutions (version 16.0).

## SUPPLEMENTARY MATERIALS TABLES AND FIGURE







## Abbreviations

CGI: CpG island
CI: confidence interval
GC: gastric cancer
GO: Gene ontology
KEGG: Kyoto Encyclopedia of Genes and Genomes
H. pylori: Helicobacter pylori
OR: odds ratio
UTR: un-translated region
TCGA: The Cancer Genome Atlas
TSS: transcription start site.

## References

[1] Schistosomes, liver flukes, Helicobacter pylori (1994). IARC Working Group on the Evaluation of Carcinogenic Risks to Humans. Lyon, 7–14 June 1994. IARC Monogr Eval Carcinog Risks Hum.

[2] Correa P (1988). A human model of gastric carcinogenesis. Cancer Res.

[3] You WC, Zhang L, Gail MH, Chang YS, Liu WD, Ma JL, Li JY, Jin ML, Hu YR, Yang CS, Blaser MJ, Correa P, Blot WJ (2000). Gastric dysplasia and gastric cancer: Helicobacter pylori, serum vitamin C, and other risk factors. J Natl Cancer Inst.

[4] Zhang L, Blot WJ, You WC, Chang YS, Kneller RW, Jin ML, Li JY, Zhao L, Liu WD, Zhang JS, Ma JL, Samloff IM, Correa P (1996). Helicobacter pylori antibodies in relation to precancerous gastric lesions in a high-risk Chinese population. Cancer Epidemiol Biomarkers Prev.

[5] You WC, Brown LM, Zhang L, Li JY, Jin ML, Chang YS, Ma JL, Pan KF, Liu WD, Hu Y, Crystal-Mansour S, Pee D, Blot WJ (2006). Randomized double-blind factorial trial of three treatments to reduce the prevalence of precancerous gastric lesions. J Natl Cancer Inst.

[6] Wong BC, Zhang L, Ma JL, Pan KF, Li JY, Shen L, Liu WD, Feng GS, Zhang XD, Li J, Lu AP, Xia HH, Lam S (2012). Effects of selective COX-2 inhibitor and Helicobacter pylori eradication on precancerous gastric lesions. Gut.

[7] Obst B, Wagner S, Sewing KF, Beil W (2000). Helicobacter pylori causes DNA damage in gastric epithelial cells. Carcinogenesis.

[8] Hmadcha A, Bedoya FJ, Sobrino F, Pintado E (1999). Methylation-dependent gene silencing induced by interleukin 1beta via nitric oxide production. J Exp Med.

[9] Shim YH, Kang GH, Ro JY (2000). Correlation of p16 hypermethylation with p16 protein loss in sporadic gastric carcinomas. Lab Invest.

[10] Chan AO, Lam SK, Wong BC, Wong WM, Yuen MF, Yeung YH, Hui WM, Rashid A, Kwong YL (2003). Promoter methylation of E-cadherin gene in gastric mucosa associated with Helicobacter pylori infection and in gastric cancer. Gut.

[11] Hu SL, Kong XY, Cheng ZD, Sun YB, Shen G, Xu WP, Wu L, Xu XC, Jiang XD, Huang DB (2010). Promoter methylation of p16, Runx3, DAPK and CHFR genes is frequent in gastric carcinoma. Tumori.

[12] Lu XX, Yu JL, Ying LS, Han J, Wang S, Yu QM, Wang XB, Fang XH, Ling ZQ (2012). Stepwise cumulation of RUNX3 methylation mediated by Helicobacter pylori infection contributes to gastric carcinoma progression. Cancer.

[13] Zouridis H, Deng N, Ivanova T, Zhu Y, Wong B, Huang D, Wu YH, Wu Y, Tan IB, Liem N, Gopalakrishnan V, Luo Q, Wu J (2012). Methylation subtypes and large-scale epigenetic alterations in gastric cancer. Sci Transl Med.

[14] Shivapurkar N, Gazdar AF (2010). DNA methylation based biomarkers in non-invasive cancer screening. Curr Mol Med.

[15] Woodson K, Mason J, Choi SW, Hartman T, Tangrea J, Virtamo J, Taylor PR, Albanes D (2001). Hypomethylation of p53 in peripheral blood DNA is associated with the development of lung cancer. Cancer Epidemiol Biomarkers Prev.

[16] Zhang Y, Su HJ, Pan KF, Zhang L, Ma JL, Shen L, Li JY, Liu WD, Oze I, Matsuo K, Yuasa Y, You WC (2014). Methylation status of blood leukocyte DNA and risk of gastric cancer in a high-risk Chinese population. Cancer Epidemiol Biomarkers Prev.

[17] Yuasa Y, Nagasaki H, Oze I, Akiyama Y, Yoshida S, Shitara K, Ito S, Hosono S, Watanabe M, Ito H, Tanaka H, Kang D, Pan KF (2012). Insulin-like growth factor 2 hypomethylation of blood leukocyte DNA is associated with gastric cancer risk. Int J Cancer.

[18] Teschendorff AE, Menon U, Gentry-Maharaj A, Ramus SJ, Gayther SA, Apostolidou S, Jones A, Lechner M, Beck S, Jacobs IJ, Widschwendter M (2009). An epigenetic signature in peripheral blood predicts active ovarian cancer. PLoS ONE.

[19] (2014). Comprehensive molecular characterization of gastric adenocarcinoma. Nature.

[20] Huang YT, Chu S, Loucks EB, Lin CL, Eaton CB, Buka SL, Kelsey KT (2016). Epigenome-wide profiling of DNA methylation in paired samples of adipose tissue and blood. Epigenetics.

[21] Davies MN, Volta M, Pidsley R, Lunnon K, Dixit A, Lovestone S, Coarfa C, Harris RA, Milosavljevic A, Troakes C, Al-Sarraj S, Dobson R, Schalkwyk LC (2012). Functional annotation of the human brain methylome identifies tissue-specific epigenetic variation across brain and blood. Genome Biol.

[22] Farre P, Jones MJ, Meaney MJ, Emberly E, Turecki G, Kobor MS (2015). Concordant and discordant DNA methylation signatures of aging in human blood and brain. Epigenetics Chromatin.

[23] Figueroa ME, Lugthart S, Li Y, Erpelinck-Verschueren C, Deng X, Christos PJ, Schifano E, Booth J, van Putten W, Skrabanek L, Campagne F, Mazumdar M, Greally JM (2010). DNA methylation signatures identify biologically distinct subtypes in acute myeloid leukemia. Cancer Cell.

[24] Noushmehr H, Weisenberger DJ, Diefes K, Phillips HS, Pujara K, Berman BP, Pan F, Pelloski CE, Sulman EP, Bhat KP, Verhaak RG, Hoadley KA, Hayes DN (2010). Identification of a CpG island methylator phenotype that defines a distinct subgroup of glioma. Cancer Cell.

[25] Bibikova M, Barnes B, Tsan C, Ho V, Klotzle B, Le JM, Delano D, Zhang L, Schroth GP, Gunderson KL, Fan JB, Shen R (2011). High density DNA methylation array with single CpG site resolution. Genomics.

[26] Shen J, Wang S, Zhang YJ, Kappil M, Wu HC, Kibriya MG, Wang Q, Jasmine F, Ahsan H, Lee PH, Yu MW, Chen CJ, Santella RM (2012). Genome-wide DNA methylation profiles in hepatocellular carcinoma. Hepatology.

[27] Shen J, Wang S, Zhang YJ, Wu HC, Kibriya MG, Jasmine F, Ahsan H, Wu DP, Siegel AB, Remotti H, Santella RM (2013). Exploring genome-wide DNA methylation profiles altered in hepatocellular carcinoma using Infinium HumanMethylation 450 BeadChips. Epigenetics.

[28] Hou L, Wang H, Sartori S, Gawron A, Lissowska J, Bollati V, Tarantini L, Zhang FF, Zatonski W, Chow WH, Baccarelli A (2010). Blood leukocyte DNA hypomethylation and gastric cancer risk in a high-risk Polish population. Int J Cancer.

[29] Kosumi K, Baba Y, Ishimoto T, Harada K, Miyake K, Izumi D, Tokunaga R, Murata A, Eto K, Sugihara H, Shigaki H, Iwagami S, Sakamoto Y (2015). Relationship between LINE-1 hypomethylation and Helicobacter pylori infection in gastric mucosae. Med Oncol.

[30] Saito M, Suzuki K, Maeda T, Kato T, Kamiyama H, Koizumi K, Miyaki Y, Okada S, Kiyozaki H, Konishi F (2012). The accumulation of DNA demethylation in Sat alpha in normal gastric tissues with Helicobacter pylori infection renders susceptibility to gastric cancer in some individuals. Oncol Rep.

[31] Oelgeschlager M, Nuchprayoon I, Luscher B, Friedman AD (1996). C/EBP, c-Myb, and PU. 1 cooperate to regulate the neutrophil elastase promoter. Mol Cell Biol.

[32] Surapureddi S, Yu S, Bu H, Hashimoto T, Yeldandi AV, Kashireddy P, Cherkaoui-Malki M, Qi C, Zhu YJ, Rao MS, Reddy JK (2002). Identification of a transcriptionally active peroxisome proliferator-activated receptor alpha -interacting cofactor complex in rat liver and characterization of PRIC285 as a coactivator. Proc Natl Acad Sci U S A.

[33] Pils D, Tong D, Hager G, Obermayr E, Aust S, Heinze G, Kohl M, Schuster E, Wolf A, Sehouli J, Braicu I, Vergote I, Van Gorp T (2013). A combined blood based gene expression and plasma protein abundance signature for diagnosis of epithelial ovarian cancer—a study of the OVCAD consortium. Bmc Cancer.

[34] Saba JD (2004). Lysophospholipids in development: Miles apart and edging in. J Cell Biochem.

[35] Huwiler A, Pfeilschifter J (2008). New players on the center stage: sphingosine 1-phosphate and its receptors as drug targets. Biochem Pharmacol.

[36] Murph M, Mills GB (2007). Targeting the lipids LPA and S1P and their signalling pathways to inhibit tumour progression. Expert Rev Mol Med.

[37] Pan KF, Zhang L, Gerhard M, Ma JL, Liu WD, Ulm K, Wang JX, Zhang Y, Bajbouj M, Zhang LF, Li M, Vieth M, Liu RY (2015). A large randomised controlled intervention trial to prevent gastric cancer by eradication of Helicobacter pylori in Linqu County, China: baseline results and factors affecting the eradication. Gut.

[38] Dixon MF, Genta RM, Yardley JH, Correa P (1996). Classification and grading of gastritis. The updated Sydney System. International Workshop on the Histopathology of Gastritis, Houston 1994. Am J Surg Pathol.

[39] Rugge M, Correa P, Dixon MF, Hattori T, Leandro G, Lewin K, Riddell RH, Sipponen P, Watanabe H (2000). Gastric dysplasia: the Padova international classification. Am J Surg Pathol.

