# Supplementary material for: Genome-wide DNA methylation profiles altered by Helicobacter pylori in gastric mucosa and blood leukocyte DNA
Source: Oncotarget. 2016 May 19;7(24):37132–44. doi: 10.18632/oncotarget.9469 (PMC5095064; doi:10.18632/oncotarget.9469)
Supplement: Supplementary file 1 [file oncotarget-07-37132-s001.pdf]

Genome-wide DNA methylation profiles altered by *Helicobacter pylori* in gastric mucosa and blood leukocyte DNA

Supplementary Materials

Supplementary Table S1: Characteristics of the subjects for methylation array detection

	Case 1	Case 2	Case 3	Case 4	Case 5	Case 6	Case 7	Case 8
Age	47	53	48	47	42	55	55	43
Sex[a]	F	M	F	M	F	M	M	M
Smoking	No	Yes	No	No	Yes	Yes	Yes	Yes
Drinking	No	No	Yes	Yes	Yes	Yes	Yes	Yes
H. pylori treatment[b]	S	S	S	S	S	S	U	U
Diagnosis before treatment	Severe CAG	Deep IM	Severe SG	Mild CAG	Severe SG	SG	Severe CAG	Mild CAG
Diagnosis after treatment	Mild CAG	Superficial IM	Mild SG	Normal	Mild CAG	SG	Mild CAG	SG

[a]F: female M: male.
[b]S: successfully eradicated U: unsuccessfully eradicated.
CAG: chronic atrophic gastritis SG: superficial gastritis IM: intestinal metaplasia.

Supplementary Table S2: Gene ontology analysis by differentially methylated CpGs from gastric mucosa. See Supplementary_Table_S2

Supplementary Table S3: KEGG pathway analysis by differentially methylated CpGs from gastric mucosa

Term	Count	%	*P* value	Benjamini
**Hypermethylated after eradication**				
B cell receptor signaling pathway	3	9.4	0.02	0.66
T cell receptor signaling pathway	3	9.4	0.039	0.66
Leukocyte transendothelial migration	3	9.4	0.046	0.57

Supplementary Table S4: Top differentially methylated CpGs and genes in gastric mucosa and blood leukocytes. See Supplementary_Table_S4

Supplementary Table S5: Characteristics of the subjects for Stage II case-control validation

		H. pylori negative *n* (%)	*H. pylori* positive *n* (%)	*P* value
Age[a]				
	< 50	10 (40.0)	14 (56.0)	0.258 [b]
	≥ 50	15 (60.0)	11 (44.0)	
Sex				
	Male	14 (56.0)	12 (48.0)	0.571[b]
	Female	11 (44.0)	13 (52.0)	
Smoking				
	Yes	8 (32.0)	4 (16.0)	0.185[b]
	No	17 (68.0)	21 (84.0)	
Drinking				
	Yes	10(40.0)	8(32.0)	0.556[b]
	No	15(60.0)	17(68.0)	
Pathology				
	SG	18(72.0)	9(36.0)	0.021[b]
	CAG	4(16.0)	13(52.0)	
	IM	3(12.0)	3(12.0)	

[a]according to the median age, subjects were divided into two groups (< 50 and ≥ 50).
[b]Pearson's χ^2 test.
CAG: chronic atrophic gastritis SG: superficial gastritis IM: intestinal metaplasia.

Supplementary Table S6: Characteristics of the subjects for Stage II self-comparison validation

		Unsuccessful eradication *n*(%)	Successful eradication *n*(%)	*P* value
Age (Mean±SD)		52.22 ± 5.56	51.92 ± 6.41	0.876[a]
Sex				
	Male	9 (69.2)	24 (64.9)	1.000[b]
	Female	4 (30.8)	13 (35.1)	
Smoking				
	Yes	4 (30.8)	18 (48.6)	0.339[b]
	No	9 (69.2)	19 (51.4)	
Drinking				
	Yes	8 (61.5)	23 (62.2)	0.968[b]
	No	5 (38.5)	14 (37.8)	
Baseline Pathology				
	SG	8 (61.5)	13 (35.1)	0.322[b]
	CAG	3 (23.1)	15 (40.5)	
	IM	2 (15.4)	9 (24.3)	

[a]*t*-test.
[b]Pearson's χ^2 test.
CAG: chronic atrophic gastritis SG: superficial gastritis IM: intestinal metaplasia.

Supplementary Table S7: Primer sequences and DHPLC denaturing temperature for candidate genes

Gene name	Primer	Sequence (5′→3′)	Product (bp)	Annealing temperature (°C)	DHPLC denaturing temperature (°C)
**Candidate genes in gastric mucosa**					
NEU1	T4-F<br>T4-R	GATTAGGAGGTTGTTAGAG<br>AATACCAATCCCTTCTTC	425	55	55
PLEKHG6	T5-F<br>T5-R	GGGTGTTTGTTATAGGTTGTT<br>CTACCCAAAATCCTATCTCA	635	56	58
PRIC285	T6-F<br>T6-R	TGTTGGTAGTATAGGAAGAGG<br>CTAACAACCAAACAAAAACTA	565	59	57.7
S1PR4	T7-F<br>T7-R	AAGAAGGTAGTTAGGGTAGGG<br>CCAACACCACCAAACA	423	60	57.0
SPI1	T9-F<br>T9-R	TGATAGTAAGTTAGGAGGGTA<br>ACAAACCTAAACCCTACAC	272	59	58.1
SYNM	T10-F<br>T10-R	ATGTTGGGATTATAGGTTTG<br>CTCCTACCCTAATCTTATCC	500	58	54.7
ARMC4	T12-F<br>T12-R	GTGGGAGGTTTATAGGGT<br>CTTCCTTTCTACTCCATTTTA	408	55	53.9
GP1BB	T14-F<br>T14-R	ATAGGAGAATAATGTTGGTG<br>AAATCCCCTCTAAATCTAAC	402	55	58.3
KANK3	T15-F<br>T15-R	GTAGGAAGGTGGGTGTTAAAG<br>CCACATTAAAAATCCCAAAT	366	55	54.8
KCNQ3	T16-F<br>T16-R	TGGGTTTTAAAGTTTTAGAGA<br>CTTCTTCCCAAAACAACA	537	55	56.5
CELSR3	T18-F<br>T18-R	GAGAAGGAGTAGGAGTATG<br>TCTATTCAACACCAAAAACAA	473	56	55.6
FOXQ1	T19-F<br>T19-R	GGTGTTTAAGGTTGAAGG<br>AACTCTACCCTAATCTTCC	388	58	53.5
**Candidate genes in blood leukocytes**					
GNAS	B5-F<br>B5-R	TAGGTTTGTAAAGGTTGG<br>CTTCCTCCTCAACTAAAA	458	52	54.7
BCOR	B8-F<br>B8-R	GGAGGAAGTTAAAATAGGTTT<br>CCCAAAACATTTTATACACAC	314	57	52.0
LTBR	B10-F<br>B10-R	GGTTGGGTTAGGGTTGTT<br>ACCCAAAACAAAAACATAAC	431	55	55.7
**Overlapped candidate genes between gastric mucosa and blood leukocytes**					
MTERF	TB2-F<br>TB2-R	GGGTTTGGGATTTTTAGT<br>TAAACAAACAAAATCTACACC	242	58	55.5

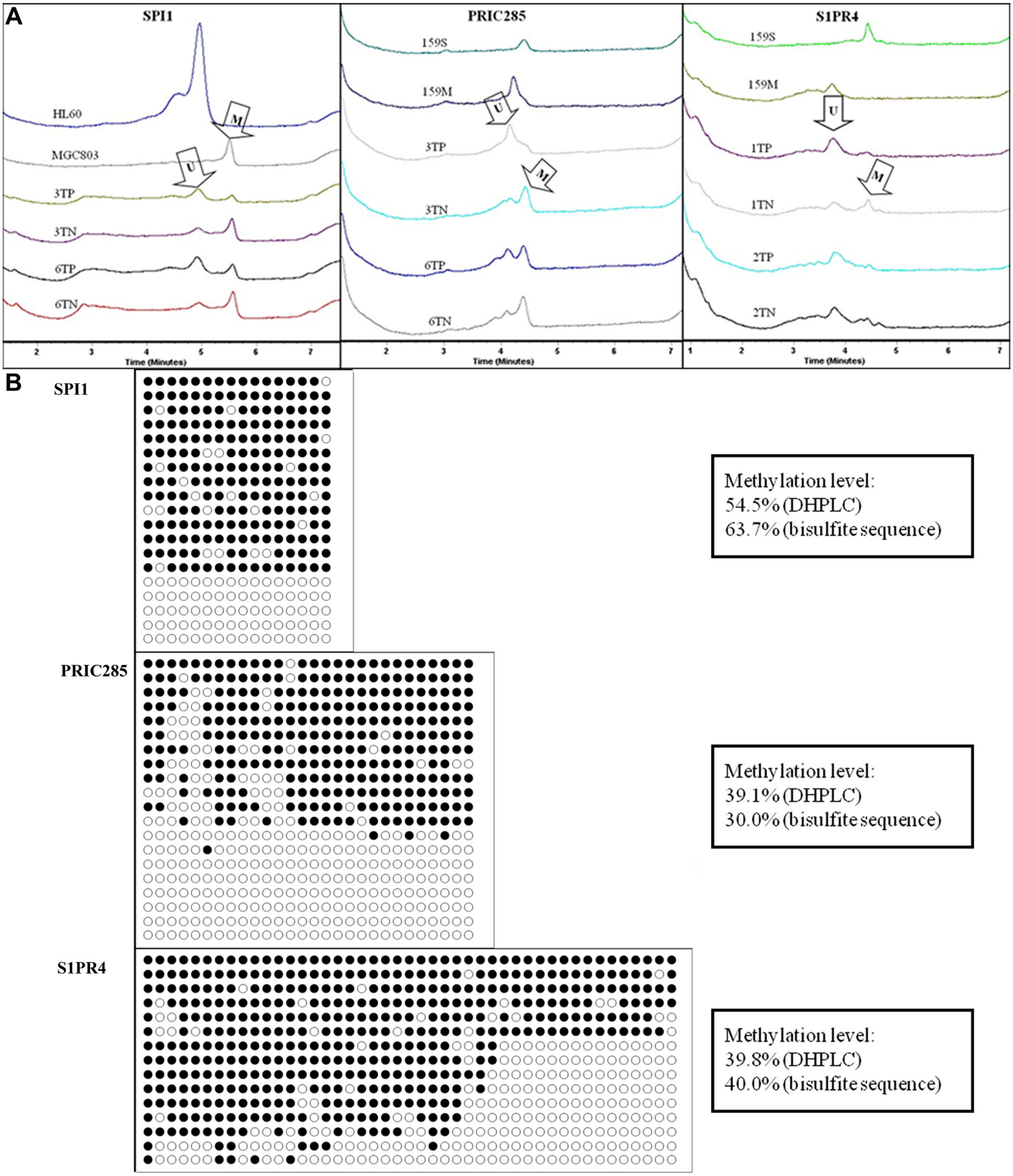

Supplementary Figure S1: Methylation detection of *SPI1, PRIC285* and *S1PR4* genes in gastric mucosa samples. (**A**) Representative DHPLC chromatograms of bisulfite PCR amplicons of *SPI1*, *PRIC285* and *S1PR4* CGIs, respectively. The methylated (M) and unmethylated (U) PCR products of each gene were separated by the DNASep analytical column at partial denaturing temperature as described in the Materials and Methods section. The peak areas corresponding to the methylated and unmethylated PCR products were used to calculate the percentage of methylated copies (methylated-peak area/total peak area) for each gene analyzed. (**B**) Representative bisulfite clone sequencing figures of *SPI1*, *PRIC285* and *S1PR4* genes and the comparison of the proportions of hypermethylated clones (> 80% CpGs were methylated in one clone) with corresponding DHPLC results of the same samples. The dark dots represent methylated CpGs.
