# Supplementary material for: Genome-wide DNA methylation profiles altered by Helicobacter pylori in gastric mucosa and blood leukocyte DNA
Source: Oncotarget. 2016 May 19;7(24):37132–44. doi: 10.18632/oncotarget.9469 (PMC5095064; doi:10.18632/oncotarget.9469)
Supplement: Supplementary file 2 [file oncotarget-07-37132-s002.docx]

Supplementary Table S2: Gene ontology analysis by differentially methylated CpGs from gastric mucosa

| **Term** | **Count** | **%** | ***P* value** | **Benjamini** |
| --- | --- | --- | --- | --- |
| **Hypomethylated after eradication** |  |  |  |  |
| tube morphogenesis | 10 | 4.8 | 1.10E-05 | 0.013 |
| tube development | 12 | 5.8 | 3.20E-05 | 0.019 |
| branching morphogenesis of a tube | 7 | 3.4 | 7.80E-05 | 0.030 |
| embryonic morphogenesis | 13 | 6.2 | 1.50E-04 | 0.043 |
| morphogenesis of a branching structure | 7 | 3.4 | 1.60E-04 | 0.038 |
| homophilic cell adhesion | 8 | 3.8 | 6.10E-04 | 0.110 |
| embryonic organ development | 9 | 4.3 | 6.30E-04 | 0.100 |
| embryonic organ morphogenesis | 8 | 3.8 | 6.70E-04 | 0.095 |
| response to hormone stimulus | 13 | 6.2 | 7.50E-04 | 0.094 |
| epithelium development | 10 | 4.8 | 9.20E-04 | 0.100 |
| chordate embryonic development | 12 | 5.8 | 1.10E-03 | 0.110 |
| embryonic development ending in birth or egg hatching | 12 | 5.8 | 1.20E-03 | 0.110 |
| response to endogenous stimulus | 13 | 6.2 | 1.70E-03 | 0.150 |
| pattern specification process | 10 | 4.8 | 2.80E-03 | 0.210 |
| cell-cell adhesion | 10 | 4.8 | 3.50E-03 | 0.240 |
| gland development | 7 | 3.4 | 3.80E-03 | 0.250 |
| sensory organ development | 9 | 4.3 | 3.90E-03 | 0.240 |
| response to steroid hormone stimulus | 8 | 3.8 | 5.40E-03 | 0.300 |
| ear morphogenesis | 5 | 2.4 | 6.00E-03 | 0.310 |
| response to estrogen stimulus | 6 | 2.9 | 6.10E-03 | 0.300 |
| regionalization | 8 | 3.8 | 6.20E-03 | 0.300 |
| regulation of transcription from RNA polymerase II promoter | 17 | 8.2 | 6.40E-03 | 0.290 |
| respiratory system development | 6 | 2.9 | 6.80E-03 | 0.300 |
| endocrine system development | 5 | 2.4 | 7.00E-03 | 0.290 |
| urogenital system development | 6 | 2.9 | 7.40E-03 | 0.300 |
| cell projection organization | 11 | 5.3 | 7.60E-03 | 0.290 |
| positive regulation of transcription from RNA polymerase II promoter | 11 | 5.3 | 8.00E-03 | 0.300 |
| neuron fate specification | 3 | 1.4 | 0.012 | 0.390 |
| tissue morphogenesis | 7 | 3.4 | 0.015 | 0.450 |
| cell fate specification | 4 | 1.9 | 0.019 | 0.520 |
| cell fate commitment | 6 | 2.9 | 0.019 | 0.520 |
| hexose metabolic process | 7 | 3.4 | 0.020 | 0.520 |
| ear development | 5 | 2.4 | 0.021 | 0.530 |
| positive regulation of transcription | 13 | 6.2 | 0.022 | 0.540 |
| cell adhesion | 15 | 7.2 | 0.022 | 0.540 |
| biological adhesion | 15 | 7.2 | 0.023 | 0.530 |
| neuron differentiation | 11 | 5.3 | 0.023 | 0.530 |
| inner ear morphogenesis | 4 | 1.9 | 0.024 | 0.530 |
| lung development | 5 | 2.4 | 0.024 | 0.520 |
| morphogenesis of an epithelium | 5 | 2.4 | 0.025 | 0.530 |
| morphogenesis of embryonic epithelium | 4 | 1.9 | 0.026 | 0.530 |
| chemical homeostasis | 12 | 5.8 | 0.026 | 0.530 |
| respiratory tube development | 5 | 2.4 | 0.026 | 0.530 |
| positive regulation of gene expression | 13 | 6.2 | 0.027 | 0.540 |
| glucose metabolic process | 6 | 2.9 | 0.027 | 0.540 |
| response to organic substance | 15 | 7.2 | 0.028 | 0.530 |
| dorsal/ventral pattern formation | 4 | 1.9 | 0.028 | 0.530 |
| positive regulation of neuron differentiation | 3 | 1.4 | 0.028 | 0.530 |
| positive regulation of macromolecule biosynthetic process | 14 | 6.7 | 0.028 | 0.500 |
| carbohydrate biosynthetic process | 5 | 2.4 | 0.030 | 0.520 |
| extracellular structure organization | 6 | 2.9 | 0.034 | 0.560 |
| monosaccharide metabolic process | 7 | 3.4 | 0.036 | 0.570 |
| epithelial tube morphogenesis | 4 | 1.9 | 0.038 | 0.580 |
| positive regulation of transcription, DNA-dependent | 11 | 5.3 | 0.038 | 0.580 |
| regulation of RNA metabolic process | 29 | 13.9 | 0.039 | 0.570 |
| beural tube development | 4 | 1.9 | 0.039 | 0.570 |
| positive regulation of cellular biosynthetic process | 14 | 6.7 | 0.039 | 0.570 |
| positive regulation of RNA metabolic process | 11 | 5.3 | 0.040 | 0.570 |
| feeding behavior | 4 | 1.9 | 0.040 | 0.570 |
| cellular carbohydrate biosynthetic process | 4 | 1.9 | 0.040 | 0.570 |
| positive regulation of cell differentiation | 7 | 3.4 | 0.041 | 0.570 |
| positive regulation of nucleobase, nucleoside, nucleotide and nucleic acid metabolic process | 13 | 6.2 | 0.043 | 0.570 |
| neural tube closure | 3 | 1.4 | 0.043 | 0.570 |
| tube closure | 3 | 1.4 | 0.043 | 0.570 |
| positive regulation of biosynthetic process | 14 | 6.7 | 0.043 | 0.570 |
| lung epithelium development | 2 | 1 | 0.042 | 0.560 |
| muscle cell differentiation | 5 | 2.4 | 0.045 | 0.570 |
| in utero embryonic development | 6 | 2.9 | 0.045 | 0.570 |
| synaptogenesis | 3 | 1.4 | 0.048 | 0.580 |
| regulation of transcription, DNA-dependent | 28 | 13.5 | 0.049 | 0.580 |
| **Hypermethylated after eradication** |  |  |  |  |
| positive regulation of immune system process | 4 | 12.5 | 0.0057 | 0.910 |
| regulation of homeostatic process | 3 | 9.4 | 0.013 | 0.940 |
| phosphate metabolic process | 6 | 18.8 | 0.015 | 0.970 |
| phosphorus metabolic process | 6 | 18.8 | 0.015 | 0.970 |
| protein amino acid phosphorylation | 5 | 15.6 | 0.018 | 0.950 |
| cell surface receptor linked signal transduction | 8 | 25 | 0.018 | 0.750 |
| immune response | 5 | 15.6 | 0.020 | 0.760 |
| regulation of lymphocyte activation | 3 | 9.4 | 0.020 | 0.730 |
| regulation of leukocyte activation | 3 | 9.4 | 0.027 | 0.760 |
| regulation of erythrocyte differentiation | 2 | 6.2 | 0.028 | 0.730 |
| regulation of cell activation | 3 | 9.4 | 0.030 | 0.720 |
| negative regulation of cytokine biosynthetic process | 2 | 6.2 | 0.031 | 0.690 |
| positive regulation of leukocyte mediated cytotoxicity | 2 | 6.2 | 0.032 | 0.680 |
| phosphorylation | 5 | 15.6 | 0.032 | 0.650 |
| positive regulation of cell killing | 2 | 6.2 | 0.037 | 0.670 |
| regulation of leukocyte mediated cytotoxicity | 2 | 6.2 | 0.038 | 0.660 |
| regulation of cell killing | 2 | 6.2 | 0.043 | 0.680 |
| positive regulation of catalytic activity | 4 | 12.5 | 0.045 | 0.680 |

Gene ontology analysis was performed using the PANTHER Classification System.
