# Supplementary material for: Genome-wide DNA methylation profiles altered by Helicobacter pylori in gastric mucosa and blood leukocyte DNA
Source: Oncotarget. 2016 May 19;7(24):37132–44. doi: 10.18632/oncotarget.9469 (PMC5095064; doi:10.18632/oncotarget.9469)
Supplement: Supplementary file 3 [file oncotarget-07-37132-s003.docx]

Supplementary Table S4: Top differentially methylated CpGs and genes in gastric mucosa and blood leukocytes

| **ID** | **Average Δβ** | ***P* value** | **Chr.** | **CpG site** | **Gene name** | **Location with gene** | **Location with CGI** |
| --- | --- | --- | --- | --- | --- | --- | --- |
| **Gastric mucosa^a^** | |  |  |  |  |  |  |
| cg01916088 | −15.69% | 0.032 | 6 | 41528531 | FOXP4 | 5′UTR | Island |
| cg15262954 | −13.21% | 0.024 | 20 | 62198872 | PRIC285(HELZ2) | 1st Exon | Island |
| cg03263514 | −11.69% | 0.037 | 22 | 19711036 | GP1BB;SEPT5 | TSS200 | Island |
| cg13510327 | −11.10% | 0.048 | 6 | 31830729 | NEU1 | TSS200 | Island |
| cg17012202 | −10.63% | 0.026 | 6 | 31830656 | NEU1 | 5′UTR | Island |
| cg16753409 | −9.76% | 0.030 | 6 | 31830896 | NEU1 | TSS200 | Island |
| cg14119437 | −9.69% | 0.034 | 6 | 31830721 | NEU1 | TSS200 | Island |
| cg26837043 | −11.00% | 0.033 | 19 | 8407675 | KANK3 | 5′UTR | Island |
| cg27121758 | −10.56% | 0.015 | 19 | 3178742 | S1PR4 | TSS200 | Island |
| cg07675031 | −10.19% | 0.012 | 11 | 47399893 | SPI1 | 1st Exon | Island |
| cg03108070 | −9.67% | 0.023 | 6 | 33245490 | B3GALT4 | 1st Exon | Island |
| cg21206147 | −9.61% | 0.034 | 2 | 23749087 | KLHL29 | 5′UTR | Island |
| cg03519157 | −9.48% | 0.014 | 11 | 46367033 | DGKZ | 5′UTR | Island |
| cg20666386 | −9.33% | 0.027 | 11 | 46367100 | DGKZ | 1st Exon | Island |
| cg20029347 | −9.13% | 0.011 | 11 | 46366877 | DGKZ | TSS200 | Island |
| cg25268678 | 15.17% | 0.025 | 12 | 6420020 | PLEKHG6 | TSS200 | Island |
| cg01096398 | 15.30% | 0.014 | 6 | 1312475 | FOXQ1 | TSS200 | Island |
| cg00366818 | 15.34% | 0.032 | 10 | 28287879 | ARMC4 | 5′UTR | Island |
| cg09140281 | 15.49% | 0.008 | 5 | 131563492 | P4HA2 | 5′UTR | Island |
| cg16544956 | 15.50% | 0.022 | 7 | 20825634 | SP8 | 5′UTR | Island |
| cg27016990 | 15.98% | 0.030 | 8 | 133492476 | KCNQ3 | 1st Exon | Island |
| cg15558982 | 15.98% | 0.041 | 1 | 115632262 | TSPAN2 | TSS200 | Island |
| cg10637512 | 16.40% | 0.002 | 15 | 99645096 | SYNM | TSS200 | Island |
| cg19104475 | 16.55% | 0.010 | 22 | 30116328 | CABP7 | TSS200 | Island |
| cg01851088 | 16.84% | 0.034 | 3 | 48700337 | CELSR3 | 5′UTR | Island |
| **Blood leukocyte^b^** | |  |  |  |  |  |  |
| cg11320369 | −11.54% | 0.034 | X | 40026673 | BCOR | 5′UTR | Island |
| cg24152605 | −8.10% | 0.005 | 19 | 57050359 | ZFP28 | 5′UTR | Island |
| cg24575067 | −7.40% | 0.016 | 20 | 44746902 | CD40 | TSS200 | Island |
| cg08564527 | −5.98% | 0.033 | 16 | 67571988 | FAM65A | 5′UTR | Island |
| cg14952966 | −5.89% | 0.004 | 11 | 125036194 | PKNOX2 | 5′UTR | Island |
| cg09896403 | −5.78% | 0.010 | 22 | 24110173 | CHCHD10 | TSS200 | Island |
| cg13259205 | −5.69% | 0.000 | 11 | 12030375 | DKK3 | TSS200 | Island |
| cg06554760 | −5.62% | 0.005 | X | 153775341 | IKBKG;G6PD | TSS200 | Island |
| cg26306994 | −5.48% | 0.001 | 12 | 6493521 | LTBR | 5′UTR | Island |
| cg21988465 | −5.22% | 0.045 | 20 | 57429277 | GNAS | 1st Exon | Island |
| cg11781306 | −5.14% | 0.001 | 5 | 140201656 | PCDHA1-5 | 1st Exon | Island |
| cg14664621 | 5.03% | 0.015 | 4 | 104640772 | TACR3 | 1st Exon | Island |
| cg15821716 | 5.84% | 0.000 | 8 | 71581642 | XKR9;LACTB2 | TSS200 | Island |
| cg01008854 | 6.02% | 0.008 | 12 | 111126998 | HVCN1 | TSS200 | Island |
| cg00801568 | 6.11% | 0.025 | 15 | 66649150 | TIPIN | TSS200 | Island |
| cg22481253 | 6.16% | 0.032 | 6 | 34204911 | HMGA1 | 5′UTR | Island |
| cg18986273 | 6.19% | 0.043 | 5 | 137673771 | FAM53C | 5′UTR | Island |
| cg21093166 | 6.26% | 0.028 | 5 | 2752145 | IRX2;C5orf38 | TSS200 | Island |

^a^Top-ranking CpGs located in promoter with the most significant methylation difference in gastric mucosa before and after *H. pylori* successful eradication.

^b^Top-ranking CpGs located in promoter with the most significant methylation difference in blood leukocytes before and after *H. pylori* successful eradication.
